# Shifting surgical strategies for osteonecrosis of the femoral head: evidence from a nationwide Japanese database

**DOI:** 10.1007/s00264-026-06772-9

**Published:** 2026-03-15

**Authors:** Hidetatsu Tanaka, Kunio Tarasawa, Yu Mori, Hiroki Kawamata, Kiyohide Fushimi, Toshimi Aizawa, Kenji Fujimori

**Affiliations:** 1 Department of Orthopaedic Surgery, Tohoku University Graduate School of Medicine, Sendai, Japan; 2 Department of Medical Information Technology Center, Tohoku University Hospital, Sendai, Japan; 3 Department of Health Policy and Informatics, Institute of Science Tokyo, Tokyo, Japan; 4 Professor Emeritus, Tohoku University, Sendai, Japan

**Keywords:** Osteonecrosis of the femora head, Diagnosis Procedure Combination, Epidemiological assessment, Total hip arthroplasty, Hip preserving surgery

## Abstract

**Introduction:**

Osteonecrosis of the femoral head (ONFH) is a progressive condition that often requires surgical intervention. Although treatment strategies have traditionally emphasized joint-preserving procedures in younger patients, advances in implant technology and perioperative management may have altered contemporary surgical decision-making. However, large-scale evidence describing temporal changes in surgical treatment patterns for ONFH is limited.

**Materials and methods:**

Using the Japanese Diagnosis Procedure Combination (DPC) database, we conducted a nationwide retrospective cohort study of patients who underwent surgical treatment for ONFH between December 2012 and March 2023. Surgical procedures were categorized as total hip arthroplasty (THA), bipolar hemiarthroplasty (BHA), proximal femoral osteotomy, pelvic osteotomy, or hip arthroscopy. Temporal trends in procedure selection were evaluated overall and by age group. Postoperative complications, including infection, deep vein thrombosis (DVT), pulmonary embolism, periprosthetic fracture, and in-hospital mortality, were compared between THA and BHA using univariate and multivariable logistic regression analyses.

**Results:**

A total of 36,109 patients were included. THA was the most frequently performed procedure throughout the study period, with its proportion increasing from 72.6% in 2012 to 90.6% in 2022, while the use of BHA and joint-preserving osteotomy steadily declined. Among patients aged ≤ 20 years, proximal femoral osteotomy predominated until 2020; thereafter, arthroplasty procedures accounted for more than half of all surgeries in this age group. Similar shifts toward THA were observed in patients aged 21–40 years. In adjusted analyses, BHA was associated with a higher risk of postoperative infection and DVT, whereas THA was associated with a higher risk of periprosthetic fracture and in-hospital mortality. No significant differences were observed in dislocation or pulmonary embolism rates.

**Conclusions:**

Nationwide data demonstrate a substantial shift in surgical management of ONFH in Japan, with increasing use of THA and declining reliance on joint-preserving procedures, even among younger patients. While arthroplasty has become the dominant treatment modality, careful consideration of long-term outcomes, complication profiles, and patient age remains essential. Integration of large-scale administrative data with detailed clinical and imaging information may further refine optimal treatment strategies for ONFH.

## Introduction

Osteonecrosis of the femoral head (ONFH) is a progressive and intractable disease characterized by impaired blood flow to the femoral head, leading to aseptic necrosis, collapse of the femoral head, and subsequent loss of joint function (1). It has been associated with various aetiologies, including trauma, glucocorticoid use, alcohol abuse, autoimmune diseases, and other factors. In Japan, ONFH frequently affects middle-aged and older working adults (2–4). Glucocorticoids are known to induce both osteoporosis and ONFH. While effective preventive strategies for glucocorticoid-induced osteoporosis, including anti-resorptive agents such as bisphosphonates and denosumab, have been established and incorporated into clinical guidelines (5, 6). ONFH substantially impairs motor function and quality of life, thereby representing a growing global health problem (1, 7, 8). A nationwide epidemiological survey conducted in Japan in 2004 estimated that approximately 11,400 patients receive treatment for ONFH annually, with around 2,200 new cases diagnosed each year (9). More than half of patients with ONFH undergo surgical intervention within 9 months of diagnosis, and spontaneous remission is not expected in many cases (10). Collapse of the femoral head results in severe hip pain, often necessitating surgical treatment such as total hip arthroplasty or osteotomy (11, 12).

Previous studies have reported that femoral head collapse in patients with ONFH can be predicted by several factors, including necrotic location, necrotic volume, and patient age (13–16), and that progression of femoral head collapse is strongly associated with the size and extent of the necrotic lesion (17–24). Treatment methods are determined based on a comprehensive assessment of the type and stage of ONFH, age, activity level, and patient preferences. Surgery has been performed when conservative treatment is limited, and joint-preserving procedures such as femoral head rotational osteotomy and varus osteotomy, or core decompression combined with bone grafting, are used (1, 12, 25–27). When conservative treatment is insufficient, joint-preserving surgical procedures, including femoral head rotational osteotomy, varus osteotomy, and core decompression combined with bone grafting, have been widely adopted, particularly in younger patients, to delay disease progression and avoid early arthroplasty (1, 12, 25–27). However, most previous studies have focused on the clinical outcomes of individual surgical procedures, while large-scale evidence capturing real-world changes in surgical decision-making over time remains limited. In particular, how treatment selection for ONFH has evolved across age groups in contemporary clinical practice remains unclear.

In recent years, treatment strategies for ONFH have evolved in response to accumulating evidence regarding long-term clinical outcomes, changing social and occupational demands, and increasing consideration of surgical invasiveness and postoperative recovery time. These factors may influence clinical decision-making and potentially shift the balance between joint-preserving surgery and joint arthroplasty in contemporary practice. However, there is limited large-scale evidence to comprehensively evaluate how evolving clinical outcomes and social factors have influenced treatment selection and surgical decision-making in patients with ONFH. Therefore, the purpose of this nationwide database study was to investigate age-stratified, real-world temporal shifts in surgical decision-making for ONFH using the Japanese Diagnosis Procedure Combination (DPC) database.

## Materials and methods

### Study design

This study was based on data obtained from the Japanese Diagnosis Procedure Combination (DPC) database and was performed in accordance with the ethical standards of the Declaration of Helsinki. Ethical approval was granted by the Institute of Science Tokyo (approval No. M2000-788) and the Tohoku University Graduate School of Medicine (approval Nos. 2021–1–1082 and 2024–1–1026). All data were retrospectively retrieved from the nationwide DPC administrative claims database. The anonymized dataset contains hospital identifiers, patient demographic characteristics, diagnoses coded using the International Classification of Diseases, 10th Revision (ICD-10), dates of admission and discharge, discharge outcomes, surgical procedures, and information on pharmaceutical use. Furthermore, the database clearly records diagnoses at admission, comorbidities present at admission, and complications that occur during hospitalization. The study period extended from January 2012 through December 2022 and encompassed a nationwide cohort of hospitals operating under the DPC system. During this period, approximately 1,100 hospitals regularly submitted clinical data and consented to their use for research purposes.

### Data selection

**Fig. 1:**
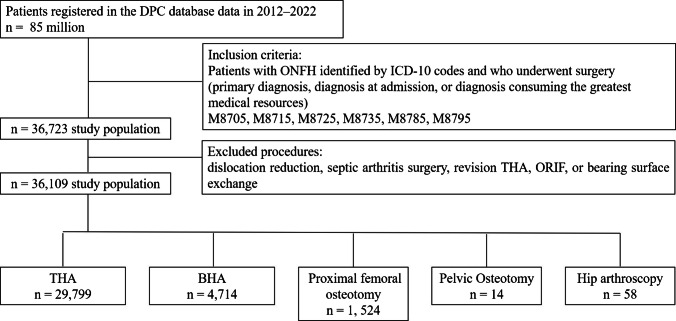
Study flow chart

Patients who underwent surgical treatment for ONFH at any of these institutions were included, enabling a comprehensive evaluation of real-world clinical practice and epidemiology of ONFH across Japan. Patients were included if the primary diagnosis, diagnosis at admission, or diagnosis consuming the greatest medical resources was ONFH, defined by the ICD-10 codes of M8705, M8715, M8725, M8735, M8785, and M8795, and if they underwent surgical treatment. Eligible surgical procedures included total hip arthroplasty (THA), bipolar hemiarthroplasty (BHA), proximal femoral osteotomy (such as femoral head rotational osteotomy and femoral head varus osteotomy), pelvic osteotomy, and hip arthroscopy. Patients were excluded if the surgical procedures performed for the diagnosis were joint dislocation reduction, surgery for septic arthritis, revision total hip arthroplasty, open reduction and internal fixation, or exchange of bearing surfaces. A total of 36,109 patients were identified and analyzed retrospectively. Baseline demographic data stratified by surgical procedure, including age, sex, body mass index (BMI), Charlson comorbidity index (CCI), and length of hospital stay, are presented in Table 1. The patient selection process is shown in Fig. 1.

**Table 1:** Baseline demographic data stratified by surgical procedure

	THA	BHA	Proximal femoral osteotomy	Pelvic Osteotomy	Scope
n	29,799	4714	1524	14	58
Age	59.9 ± 14.8	71.3 ± 16.0	33.5 ± 11.0	21.2 ± 9.2	43.4 ± 15.2
Gender (%)					
Men	48.1	31.8	64.0	7.1	58.6
Women	51.9	68.2	36.0	92.9	41.4
BMI	23.3 ± 5.9	21.9 ± 4.1	23.4 ± 11.3	22.2 ± 5.0	23.5 ± 3.8
CCI	0.78 ± 1.09	0.97 ± 1.22	0.40 ± 0.77	0.43 ± 0.65	0.48 ± 0.82
Length of hospital stay	26.6 ± 17.2	35.8 ± 25.7	48.9 ± 26.8	40.9 ± 27.4	26.4 ± 24.2

Age, BMI, CCI, and length of hospital stay are shown as mean ± standard deviation.

*BMI* Boby Mass Index, *CCI* Charlson Comobidity Index, *THA* Total Hip Arthroplasty, *BHA* Bipolar hemiarthroplasty.

Outcomes of interest included the number and proportion of each surgical procedure, which were summarized by year and by age group, and temporal trends were evaluated. In addition, complication rates, including dislocation, infection, deep vein thrombosis (DVT), and pulmonary embolism (PE), were compared between THA and BHA.

### Statistical analysis

All data are presented as means with standard deviations. Differences between the two groups were assessed using the χ^2^ test for categorical variables and Student's t-test for continuous variables, as appropriate. The Shapiro–Wilk test was used to assess normality. Univariate and multivariate logistic regression analyses were performed to examine the complications associated with THA and BHA. All statistical tests were two-tailed, and P-values < 0.05 were considered statistically significant. All analyses were performed using JMP version 18.0 (SAS, Cary, North Carolina, USA).

## Results

### Temporal trends in the distribution of surgical procedures

**Fig. 2:**
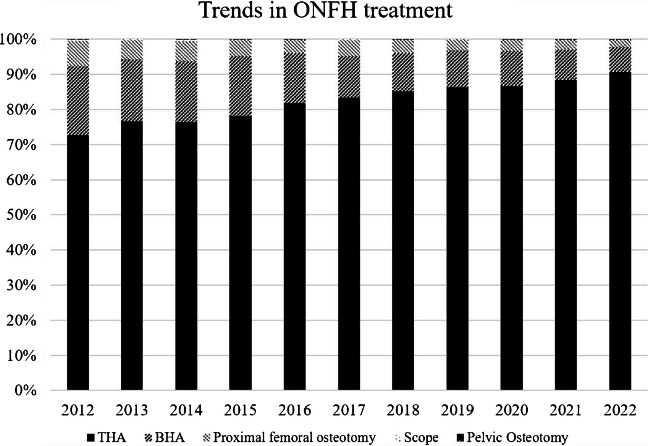
Trends in ONFH treatment (overall population). Temporal trends in surgical treatment strategies for ONFH from 2012 to 2022 in the overall study population. The proportions of THA, BHA, proximal femoral osteotomy, pelvic osteotomy, and arthroscopic procedures are presented as percentages of total surgical procedures performed each year

Between 2012 and 2022, the annual number of surgical procedures for ONFH ranged from 2,587 to 3,531 cases. The most commonly performed procedure was THA throughout the study period, accounting for an increasing proportion of all surgeries (Fig. 2). Specifically, THA represented 72.6% of procedures in 2012 (1,879 of 2,587 cases) and increased to 90.6% in 2022 (2,864 of 3,161 cases). In contrast, the use of BHA steadily declined over time, both in absolute numbers and as a proportion, decreasing from 508 cases (19.6%) in 2012 to 225 cases (7.1%) in 2022. Similarly, proximal femoral osteotomy showed a continuous reduction, accounting for 7.3% of procedures in 2012 (189 cases) but only 2.2% in 2022 (70 cases). Pelvic osteotomy and hip arthroscopy were rarely performed during the study period and together accounted for less than 1% of all procedures in most years. Pelvic osteotomy remained infrequent without a clear temporal trend, while hip arthroscopy showed a gradual decline and was no longer performed after 2020 (Fig. 2).

### Temporal changes in surgical procedure selection among younger patients

**Fig. 3:**
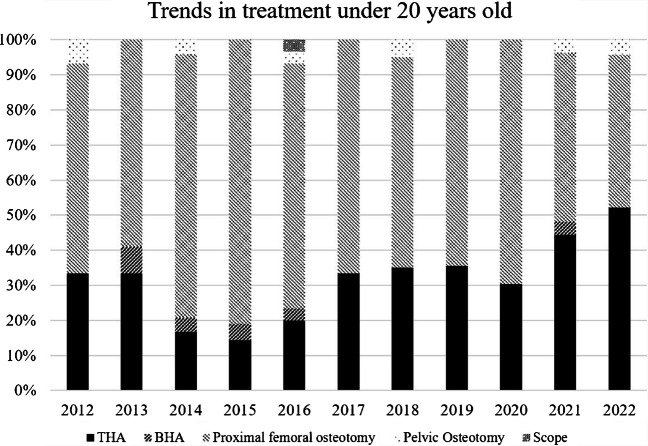
Trends in treatment in patients aged ≤ 20 years. Annual distribution of surgical procedures for ONFH in patients aged ≤ 20 years between 2012 and 2022. Although joint-preserving procedures predominated in earlier years, the proportion of arthroplasty procedures increased in recent years

**Fig. 4:**
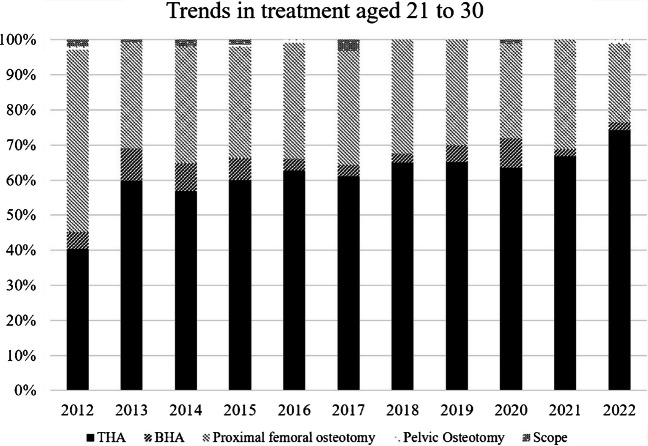
Trends in treatment in patients aged 21–30 years. Temporal trends in surgical management of ONFH in patients aged 21–30 years. A progressive increase in THA was observed over time, accompanied by a relative decline in joint-preserving osteotomy procedures

**Fig. 5:**
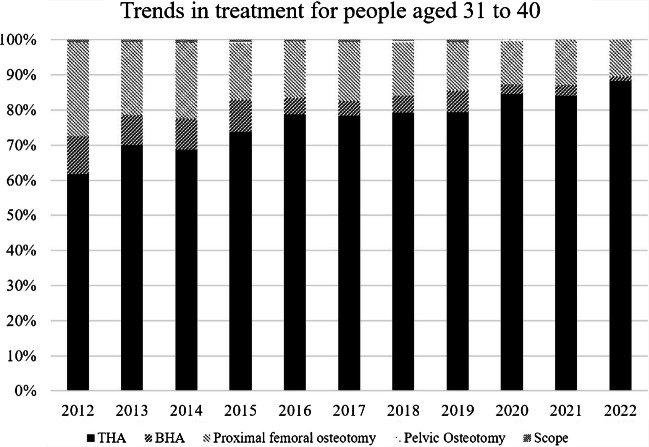
Trends in treatment in patients aged 31–40 years. Changes in surgical treatment patterns for ONFH in patients aged 31–40 years from 2012 to 2022. THA consistently accounted for the majority of procedures, with a gradual increase over the study period

Among patients aged ≤ 20 years, joint-preserving procedures, particularly proximal femoral osteotomy, accounted for the majority of surgical treatments during the early study period (Fig. 3). From 2012 to 2020, proximal femoral osteotomy consistently accounted for more than half of all procedures in this age group, ranging from 60–71% annually. In contrast, arthroplasty procedures were less frequently performed, with THA and bipolar BHA together accounting for approximately 30–40% of procedures during this period. However, a clear shift in surgical strategy was observed after 2021. In patients aged ≤ 20 years, the proportion of proximal femoral osteotomy decreased markedly from 69.7% in 2020 (23 of 33 cases) to 48.1% in 2021 (13 of 27 cases) and further to 43.5% in 2022 (10 of 23 cases). Correspondingly, the proportion of arthroplasty procedures increased, with THA and BHA accounting for 48.1% of procedures in 2021 and 52.2% in 2022, surpassing joint-preserving procedures in the most recent years. In patients aged 21–30 years, THA was the most frequently selected procedure throughout the study period, accounting for approximately 45–60% of surgeries annually (Fig. 4). Proximal femoral osteotomy remained a substantial component in this age group but demonstrated a gradual decline in proportion over time, decreasing from 51.4% in 2012 to 22.6% in 2022. BHA, pelvic osteotomy, and hip arthroscopy were infrequently performed and together accounted for less than 10% of procedures in most years. Similarly, in patients aged 31–40 years, THA consistently predominated, accounting for approximately 60–75% of all procedures across the study period (Fig. 5). The proportion of proximal femoral osteotomy steadily declined from 26.9% in 2012 to 10.6% in 2022, while BHA and other procedures remained uncommon.

### Comparison of postoperative complications between THA and BHA

Baseline demographic characteristics differed significantly between patients undergoing THA and BHA (Table 1). Patients treated with BHA were significantly older than those treated with THA (71.3 ± 16.0 vs. 59.9 ± 14.8 years, *p *< 0.05) and had a higher comorbidity burden, as reflected by a greater CCI (0.97 ± 1.22 vs. 0.78 ± 1.09, *p *< 0.05). In addition, BMI was lower in the BHA group (21.9 ± 4.1 vs. 23.3 ± 5.9, *p *< 0.05), and the length of hospital stay was significantly longer compared with the THA group (35.8 ± 25.7 vs. 26.6 ± 17.2 days, *p *< 0.05). The results of the pairwise comparison of postoperative complications between THA and BHA are summarized in Table 2. In univariate analyses, significant differences were observed between the two procedures with respect to postoperative infection, periprosthetic fracture, DVT, and in-hospital mortality (*p *< 0.05 for all). These differences remained statistically significant after multivariable adjustment. Specifically, BHA was associated with a higher risk of postoperative infection (adjusted odds ratio [OR], 1.45; 95% confidence interval [CI], 1.028–2.045; *p *< 0.05) and DVT (adjusted OR, 2.309; 95% CI, 1.873–2.872; *p *< 0.05). Conversely, BHA was associated with a lower risk of periprosthetic fracture (adjusted OR, 0.473; 95% CI, 0.317–0.706; *p *< 0.05) and in-hospital mortality (adjusted OR, 0.237; 95% CI, 0.145–0.387; *p *< 0.05) compared with THA. No significant differences were observed between THA and BHA with regard to postoperative dislocation, reoperation, or pulmonary embolism in either univariate or multivariable analyses.

**Table 2:** Pairwise comparison of postoperative complications between THA and BHA

Complications			Univariate analysis			Multivariable analysis		
	n	OR	95% CI	P-value	OR	95% CI	χ2 statics	P-value
Dislocation	345	1.080	0.788–1.488	0.694	1.201	0.783–1.865	0.736	0.391
Infection	379	1.424	1.017–1.994	< 0.05	1.45	1.028–2.045	4.89	< 0.05
Periprosthetic fracture	146	0.464	0.329–0.675	< 0.05	0.473	0.317–0.706	12.09	< 0.05
Reoperation	667	0.934	0.755–1.169	0.569	0.914	0.667–1.253	0.31	0.578
DVT	1410	2.325	1.878–2.879	< 0.05	2.309	1.873–2.872	75.21	< 0.05
PE	67	1.088	0.478–2.126	0.999	1.018	0.477–2.173	0.002	0.964
Mortality during hospitalization	67	0.233	0.143–0.381	< 0.05	0.237	0.145–0.387	28.3	< 0.05

*P*-values of < 0.05 are considered significant by the χ2 test.

*OR* Odds Ratio, *CI* Confidence Interval, *DVT* Deep Vein Thrombosis, *PE* Pulmonary Embolism.

## Discussion

In this nationwide study using the Japanese Diagnosis Procedure Combination (DPC) database, we comprehensively analyzed the real-world surgical management of osteonecrosis of the femoral head (ONFH), focusing on temporal trends in procedure selection and postoperative complications according to surgical technique. Consistent with previous epidemiological reports, ONFH predominantly affected middle-aged and older adults in Japan (1, 3, 10, 28). The most notable finding of the present study was a substantial shift in surgical strategy over the past decade, characterized by a marked increase in the use of THA and a corresponding decline in BHA and joint-preserving osteotomy procedures. This study uses DPC data and provides detailed data on background and comorbidities. Combined with previous studies on degenerative diseases, rheumatoid arthritis, hip fractures, trends and regional variations, and epidemics, this study deepens our understanding of broader trends and characteristics of orthopaedic care in Japan (29–35).

The increasing preference for THA likely reflects advances in implant design, bearing materials, and minimally invasive surgical techniques, which have collectively improved short- to mid-term clinical outcomes while reducing perioperative morbidity and facilitating faster postoperative recovery. These technological and procedural developments may have lowered the threshold for selecting THA even in relatively younger patients, particularly in those with advanced symptoms or high functional demands (36, 37). In contrast, although BHA has traditionally been considered a treatment option for patients with ONFH at stage 3 or earlier, accumulating evidence has raised concerns regarding persistent postoperative pain, acetabular cartilage degeneration due to outer head migration, and inferior long-term functional outcomes (38, 39). These limitations, together with the relatively high conversion rates to THA reported in long-term follow-up studies, have likely contributed to the declining use of BHA in contemporary clinical practice (40). Nevertheless, BHA may still be selected in specific clinical contexts, such as in older patients with low activity demands, poor bone quality, or when minimizing surgical invasiveness is prioritized. These factors may partly explain why BHA continues to be performed despite its overall declining trend.

Age-stratified analyses revealed clear differences in surgical selection patterns across generations. In patients aged ≤ 20 years, joint-preserving procedures—primarily proximal femoral osteotomy—had historically been the dominant treatment strategy. However, since 2021, the proportion of arthroplasties has approached, and in some cases exceeded, that of osteotomies in this age group, suggesting a potential shift away from the long-standing principle of prioritizing joint preservation in younger patients in real-world clinical practice. A similar but more pronounced transition from osteotomy to arthroplasty was observed in patients aged 21–40 years, indicating a generational trend toward earlier adoption of prosthetic reconstruction. However, these findings should be interpreted with caution, as the number of patients in this age group was relatively small, particularly in recent years.

These findings are consistent with previous registry-based and population-level studies that have reported evolving surgical treatment patterns for ONFH. Nationwide database analyses from East Asia have demonstrated an increasing proportion of arthroplasty procedures, particularly THA, relative to joint-preserving osteotomies and decompression techniques over recent years, reflecting a broader shift away from traditional joint-preserving strategies toward prosthetic reconstruction even in younger patients (13, 41). Moreover, recent cohort studies have reported favourable long-term outcomes of THA in patients under 30 years of age, further supporting the expanding use of arthroplasty in younger populations in whom joint preservation was previously prioritized (42). However, these findings should be interpreted cautiously given the relatively small sample size in this age group, particularly in recent years.

Despite these trends, joint-preserving surgery remains an important therapeutic option, particularly for young patients, when considering the long-term durability of implants and the potential need for revision surgery after THA. Previous studies have demonstrated favorable mid- to long-term outcomes of osteotomy-based procedures for appropriately selected patients with ONFH (43–46). Moreover, THA performed for ONFH has been reported to be associated with higher complication rates compared with THA for primary osteoarthritis, underscoring the importance of careful patient selection and long-term treatment planning (47, 48). In this context, future advances in regenerative medicine and biologic therapies may further expand treatment options and help bridge the gap between joint preservation and arthroplasty (49, 50).

In an analysis of postoperative complications, BHA was associated with significantly higher rates of infection and DVT compared with THA, whereas THA was associated with higher rates of periprosthetic fracture and in-hospital mortality. These findings may reflect differences in patient background characteristics and surgical indications between the two procedures. Patients undergoing BHA were significantly older and had a higher comorbidity burden, which may partly explain the increased risks of infection and thromboembolic events. Conversely, the higher rates of periprosthetic fracture and in-hospital mortality observed in THA may be related to technical complexity and procedure-related factors. The observed associations should not be interpreted as causal, given residual confounding by indication inherent to administrative data. These results emphasize the importance of meticulous perioperative management, including infection control measures and thromboprophylaxis, especially in patients undergoing THA for ONFH.

Large-scale epidemiological studies using the DPC database are expected to play an increasingly important role in orthopaedic research, contributing to the establishment of standard treatment strategies and the development of evidence-based clinical guidelines (29–34, 47). Nevertheless, several limitations inherent to the DPC database should be acknowledged. First, detailed clinical information such as imaging findings, extent of necrosis, and disease stage is not available, precluding more granular risk stratification. Second, patient preferences, surgeon experience, and institutional factors influencing surgical decision-making cannot be assessed. In addition, although the DPC system covers approximately 70% of acute-care hospitals in Japan, data from non-participating institutions are not included, which may limit generalizability. Future studies integrating administrative claims data with imaging findings, clinical staging, and patient-reported outcome measures will be essential to establish more refined, patient-centered treatment strategies for ONFH and to optimize long-term outcomes across different age groups. In addition, residual confounding by indication may persist despite multivariable adjustment, as factors such as frailty, bone quality, and surgeon preference could not be fully accounted for in the administrative database. Therefore, the observed differences in postoperative complications between THA and BHA should be interpreted as associations rather than causal effects.

## Conclusion

In this nationwide analysis using the Japanese DPC database, we identified a marked transition in surgical treatment strategies for ONFH over the past decade. The use of THA has increased substantially, while BHA and joint-preserving osteotomy procedures have declined across all age groups, including adolescents and young adults. Although advances in arthroplasty techniques have expanded indications for THA, joint-preserving surgery remains an important option for selected younger patients when long-term implant durability is considered. Differences in complication profiles between THA and BHA underscore the need for individualized surgical decision-making and optimized perioperative management. Future studies combining administrative databases with imaging findings and patient-reported outcomes are warranted to establish more precise, age- and stage-specific treatment algorithms for ONFH.

## Data Availability

No datasets were generated or analysed during the current study.

## Abbreviations

ONFH: Osteonecrosis of the femoral head
DPC: Diagnosis Procedure Combination
THA: Total hip arthroplasty
BHA: Bipolar hemiarthroplasty
DVT: Deep vein thrombosis
BMI: Body mass index
CCI: Charlson comorbidity index
OR: Odds ratio
CI: Confidence interval
